# A new protein-ligand binding sites prediction method based on the integration of protein sequence conservation information

**DOI:** 10.1186/1471-2105-12-S14-S9

**Published:** 2011-12-14

**Authors:** Tianli Dai, Qi Liu, Jun Gao, Zhiwei Cao, Ruixin Zhu

**Affiliations:** 1 College of Life Science and Biotechnology, Tongji University, 200092, Shanghai, China; 2 College of Information Engineering, Shanghai Maritime University, 201306, Shanghai, China; 3 Shanghai Center for Bioinformation and Technology, 100 Qinzhou Road, Shanghai, 200235, China; 4 Department of Chinese Material Medica, Liaoning University of Traditional Chinese Medicine, Shenyang, Liaoning 110032, China

## Abstract

**Background:**

Prediction of protein-ligand binding sites is an important issue for protein function annotation and structure-based drug design. Nowadays, although many computational methods for ligand-binding prediction have been developed, there is still a demanding to improve the prediction accuracy and efficiency. In addition, most of these methods are purely geometry-based, if the prediction methods improvement could be succeeded by integrating physicochemical or sequence properties of protein-ligand binding, it may also be more helpful to address the biological question in such studies.

**Results:**

In our study, in order to investigate the contribution of sequence conservation in binding sites prediction and to make up the insufficiencies in purely geometry based methods, a simple yet efficient protein-binding sites prediction algorithm is presented, based on the geometry-based cavity identification integrated with sequence conservation information. Our method was compared with the other three classical tools: PocketPicker, SURFNET, and PASS, and evaluated on an existing comprehensive dataset of 210 non-redundant protein-ligand complexes. The results demonstrate that our approach correctly predicted the binding sites in 59% and 75% of cases among the TOP1 candidates and TOP3 candidates in the ranking list, respectively, which performs better than those of SURFNET and PASS, and achieves generally a slight better performance with PocketPicker.

**Conclusions:**

Our work has successfully indicated the importance of the sequence conservation information in binding sites prediction as well as provided a more accurate way for binding sites identification.

## Background

Proteins are the material basis of all life, the key components of body cells, and play important roles in the process of life activity. Since in most cellular processes, proteins interact with other molecules to perform their biological functions, the successful identification of ligand-binding sites on protein surfaces becomes vital and necessary to explore the proteins comprehensively [1]. In addition, as a result of various structural genomics projects performed, structural information of proteins with little or no functional annotations has been explosively increasing. Such increasingly accumulated data have become to attract much more interests in exploring the relationship between protein structure and function as well as elucidating the functions from their structures rather than merely from sequences.

In recent decades, many computational methods have been developed for candidate binding sites identification. Briefly, these algorithms can be divided into three categories, i.e.(1) purely geometry based methods, which follow the assumption that the protein-ligand binding sites are generally located at crevices on the protein surface or cavities in the protein. When the shapes of protein surface were calculated, it can be easily to predict the candidate protein-ligand binding sites without any ligands information. Methods following in this category include POCKET [2], LIGSITE [3], PASS [4], SURFNET [5] and, PocketPicker [6] etc. It is worth noting that this kind of methods focuses only on the shapes of protein surface without considering the physicochemical properties of amino acids. What’s more, a major number among these algorithms are based on the cubic grid representation, which means that their following results are often protein orientation dependent; (2) energetic based methods, which coat the protein surface with a layer of probes to calculate van der Waals interaction energies between the protein and probes. The energetically favorable probe sites are clustered according to their spatial proximity. Then the identified clusters are ranked according to the sum of the interaction energies within each cluster. As an example, Q-SiteFinder [7] is a classical tool following in this category; (3) knowledge based methods, which including various statistical methods [8], machine learning methods [9] and similarity comparison method set. Besides, a part of them predict protein-ligand binding sites by searching for clusters or patterns of conserved residues [10,11]. These method stake the assumption that the residues located in protein-ligand binding site usually being more important and more highly conserved than those located in other parts through evolution. Although the results for certain methods with only sequence conservation information are not satisfactory [12,13], it is still expected to be helpful in re-ranking the pockets in the process of prediction [14].

In summary, in this study, in order to investigate the contribution of sequence conservation information in binding sites prediction and to make up the insufficiencies for purely geometry based methods, we aims at designing a simple, yet efficient and practical binding site prediction algorithm based on the integration of sequence conservation information with geometry-based cleft identification.

## Methods

### Algorithm workflow

**Figure 1:**
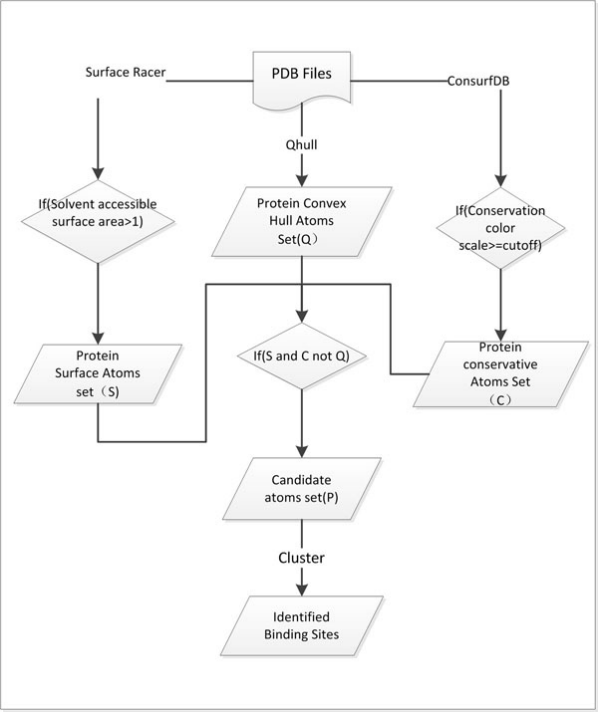
**The flowchart of proposed algorithm.** Overview of our method. The prediction is based on the geometry-based cavity identification integrated with sequence conservation information.

An overview of our method is shown in Figure 1. It is composed of three steps: (1) Calculation of geometrical characteristics of protein (cleft identification); (2) Filtering with sequence conservation information, and (3) Clustering potential atoms which will form the prediction binding sites according to their spatial distance-based similarity.

Step 1: for each protein structure, the solvent accessible surface area (SASA) of each atom is calculated first. The SASA values can be available from many tools such as NACCESS [15], ASC [16], Surface Racer [17] etc. Here Surface Racer is used because it can exactly calculate accessible surface area on most common computer platforms. An atom is considered as an interface alone if its SASA is over 1Å [18]. In our study, these atoms are denoted as Set S (Surface). In addition, Qhull [19] is applied to calculate the convex hull based on all the protein atoms [20,21]. Considering the convex hull obtained here is just a smallest convex set of atom points, it is expanded by adding atoms which are within certain distance from the origin convex atoms (the parameter is adjustable, 6.5Å is used here). Such new convex set is denoted as Set Q (Qhull). Finally, after calculating those atoms which included in Set S and excluded from Set Q, it is convenient to get a set of protein atoms which locate at protein’s crevice regions.

Step 2: since the first step just identifies the clefts on the protein surface without any biological significance, the sequence conservation information are further added as a filter [8,14,22] to curate our results. This is achieved by the ConSurf-DB [23] which provides the pre-calculated evolutionary conservation profiles for proteins with known structures in the PDB. In ConSurf-DB, every residue in every corresponding protein is evaluated with a normalized conservation score. And then the normalized scores are binned into the 1-9 color scales for representing the conservation grades and projected on the 3D model of the query protein, where 1 corresponds to maximal variability and 9 to maximal conservation [23]. It is important to note that although the same color scale in ConSurf-DB is used in all the protein families, the conservation scores are not absolute and hence, defining the conservation scores as a filter between different protein families might be misleading. Accordingly, in this study, ConSurf-DB results are interpreted using the color scales rather than the conservation scores, and only the residues greater than or equal to certain conservation grade cutoff (such as 7, 8, 9. 7 is used here) will be retained [13]. We denote all the atoms of those conservative residues as Set C (Conservative atoms). After such physicochemical property as a biological factor, those atoms which appear on protein’s clefts will accordingly hold the sequence conservation information. These atoms are denoted as Set P (Potential atoms).

Step 3: a simple hierarchical clustering algorithm is applied to cluster those potential atoms according to their spatial distances. Each output cluster stands for a presumed protein-ligand binding site, and the center of each cluster represents the geometric center of each binding site. Geometric centers within a certain distance threshold (8Å used here) are grouped together as a new cluster [24] whereas the corresponding geometric center should be recalculated.

Besides the candidate protein-ligand binding site identification itself, binding site ranking is also a very important tissue. For instance, since there are often several presumed binding sites that can be detected on a protein surface, in order to select the more relevant ones, it is necessary to derive an approach to characterize and rank them. It is often said that the largest pocket tends to frequently correspond to the observed ligand binding site [25]. Based on this assumption, a most number of prediction methods rank the candidate sites by pocket size. On the other hand, different studies have tried to solve this ranking problem from other perspectives [14,26,27]. Among them, evolutionary information such as sequence conservation has been shown to be successful for re-ranking the binding sites [14]. Therefore, in our study, the candidate binding sites are ranked according to the conservation score of all residues in the same cluster.

### Test dataset

**Figure 2:**
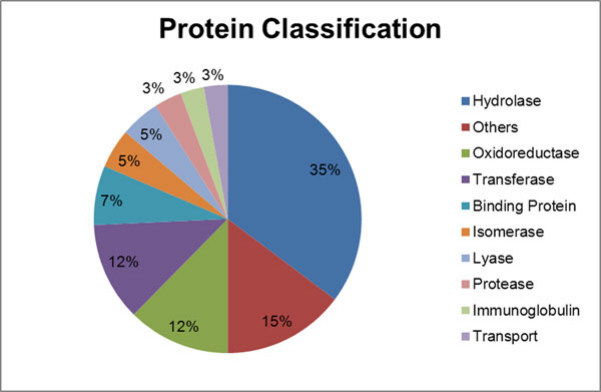
**Distribution of the protein dataset by molecular function**. This is a rough statistics on the protein dataset classification.

In this study, a regularly used dataset [14] is chosen as the standard test data, which consists of 210 non-duplicated protein-ligand complexes derived from the Protein Ligand Database (PLD) [28]. A rough statistics on the protein dataset classification has been shown in Figure 2. Furthermore, in order to assess the binding site prediction performance of our method, the identified sites are needed to be compared to the real binding sites. For the 210 bound proteins, the ligands are taken away when making predictions and then put back when performing evaluation. The PocketPicker criterion (PPc) [6] is adopted as the prediction criterion in this study. It defines the prediction to be a hit if the geometric center of the presumed binding site is within 4Å from any atom of the ligand.

## Results and discussion

**Figure 3:**
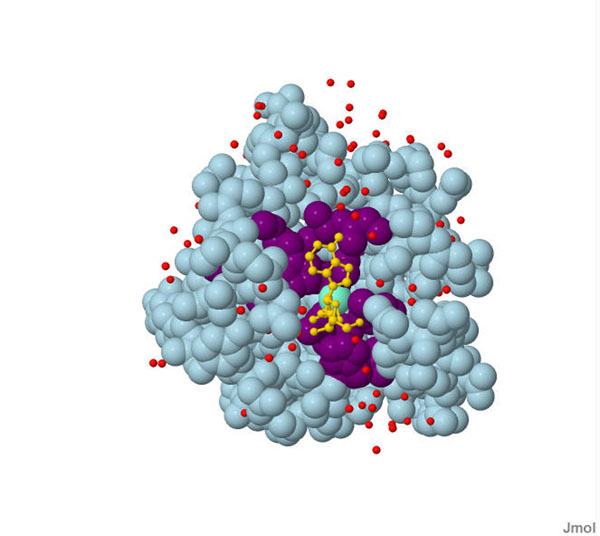
**One case study of our method.** PDB ID: 6RNT. (Red points: water molecule; Light blue: the whole protein; Golden: ligand molecular; Aquamarine : binding site’s center and Purple: predicted binding site constituted by amino acids.)

Our algorithm is tested on 210 protein-ligand complexes. The presumed protein-ligand binding sites are ranked by conservation score. A successful prediction example [PDB: 6RNT] [29] visualized with Jmol [30] is presented in Figure 3. In order to check the contribution of sequence conservation as well as the feasibility of our method, three purely geometry based methods i.e. PocketPicker, SURFNET, and PASS with their own ranking methods are also tested for comparison [6,24]. The accuracy of the first one (TOP 1) and first three (TOP 3) in the prediction ranking lists have been calculated. It is indicated from Table 1 that our method obtained a 59% success rate for the top one prediction which means almost 124 of the 210 proteins are correctly predicted. The top one result is much higher than that of SURFNET and PASS. Although the top three success rate seems to be a little bit worse than PASS, our method still performs better than others. On the whole, the success rate in our study is comparable to that of PocketPicker which is one of the most popular prediction tools presented in 2007 while our method with the quick-reading operating process and grid-presentation independent.

**Table 1:** Prediction success rate presented by different binding-sites prediction methods

Methods	TOP1	TOP3
Conservation score	59%	75%
Volume	45%	63%
SURFNET(Control)	42%	57%
PASS(Control)	51%	80%
PocketPicker(Control)	59%	71%

As small molecular ligands are tend to combine with proteins in larger cavities on protein surface, the volume can be used as a ranking method to choose the likely candidates. In this study, the candidate binding sites are also ranked according to the space volume. The success rates are listed in the Volume column in Table 1. It can be seen that this kind of ranking method doesn’t show any advantage to that by conservation score. What’s more, the top one success rate ranked by volume hardly achieves 50%. It indicates that such volume ranking rule can’t be generalized with its own limit.

**Figure 4:**
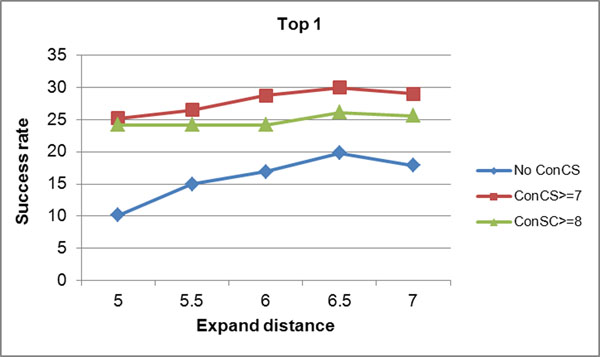
**TOP1 Success rates achieved by setting different parameters.** The accuracy of the first one pocket sites (TOP 1) in the prediction ranking lists was different under different parameter combinations.

**Figure 5:**
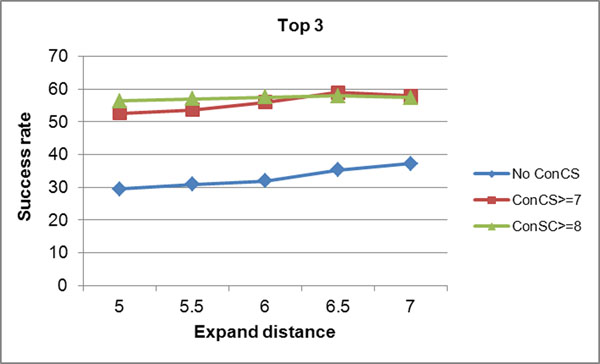
**TOP3 Success rates achieved by setting different parameters.** The accuracy of the first three pocket sites (TOP 3) in the prediction ranking lists was different under different parameter combinations.

In addition, two factors, i.e. expand distance from origin convex hull set and the conservation color scale (ConCS) are tested for their influence in our study. The top one and top three success rates under different combinations of these two factors are shown in Figure 4 and Figure 5, respectively. The success rates are derived according to the cavities’ space volume in a sequential manner. It can be seen that no matter how the conservation score is set, the expand distance at 6.5 Å always performs a better success rate. The data of ConCS>=9cannot be available because when under such special condition, the number of the candidate atoms in some protein structures will be too small to form predicted protein-ligand binding sites. Moreover, compared to those candidate atoms without evolutionary information (the No ConCS line), we find that when the sequence conservation is introduced as a filter, the success rates in top 1 and top 3 are all improved significantly. However, it also can be seen that the higher conservation color scale the candidate atoms obtain doesn’t often result the higher prediction accuracy (the red line tends to be above the green line). We explained this by that the atoms forming the ligand binding sites are not only the most conservative ones but also the relatively conservative ones, which should be validated further more.

In summary, our study has validated the insufficiency of purely geometric methods, and at the same time, reflected the significance of sequence conservation in ligand binding sites prediction.

## Conclusions

The prediction of protein-ligand binding sites has great significance for the protein function annotation and computer-aided drug design. Though many different outstanding studies have been carried out to solve this problem, some of them just use complicated calculation methods based on protein shape descriptor rather than considering other physicochemical and sequence properties with biological characteristics. In this paper, a simple yet efficient binding site prediction algorithm is designed based on the integration of geometry and sequence conservation information. The algorithm is tested on a regularly used benchmark dataset, and shows an encouraging result with the success rates come to 59% and 75% for the top one and top three pocket sites, respectively. Our algorithm performs comparative to PocketPicker while with more convenient prediction procedure. Last but not least, our result also reflects the un-ignorable importance of sequence conservation information which can be an effective attribute in ligand binding site prediction.

## Competing interests

The authors declare that they have no competing interests.

## Authors’ contributions

Conceived and designed the experiments: TD, ZC, RZ. Performed the experiments: TD, ZC, RZ. Analyzed the data: TD, QL, JG, ZC, RZ. Wrote the first draft: TD. Revised the draft: TD, QL, JG, ZC, RZ.
